# Peripapillary Retinal Nerve Fiber Layer Vascular Microcirculation in Glaucoma Using Optical Coherence Tomography–Based Microangiography

**DOI:** 10.1167/iovs.15-18909

**Published:** 2016-07-21

**Authors:** Chieh-Li Chen, Anqi Zhang, Karine D. Bojikian, Joanne C. Wen, Qinqin Zhang, Chen Xin, Raghu C. Mudumbai, Murray A. Johnstone, Philip P. Chen, Ruikang K. Wang

**Affiliations:** 1 Department of Bioengineering, University of Washington, Seattle, Washington, United States; 2 Department of Ophthalmology, University of Washington, Seattle, Washington, United States; 3 Department of Ophthalmology, Beijing Anzhen Hospital, Capital Medical University, Beijing, China

**Keywords:** optical coherence tomography angiography, optical microangiography, glaucoma, retinal nerve fiber layer, perfusion

## Abstract

**Purpose:**

To investigate the vascular microcirculation changes in the retinal nerve fiber layer (RNFL) in normal, glaucoma suspect, and open-angle glaucoma (OAG) groups using optical coherence tomography–based microangiography (OMAG).

**Methods:**

One eye from each subject was scanned with a Cirrus HD-OCT 5000–based OMAG prototype system montage scanning protocol centered at the optic nerve head (ONH). Blood flow signals were extracted using OMAG algorithm. Retinal nerve fiber layer vascular microcirculation was measured by calculating the blood flux index and vessel area density within a 1.2-mm width annulus centered at the ONH with exclusion of big retinal vessels. One-way ANOVA were performed to analyze the RNFL microcirculation among groups. Linear-regression models were constructed to analyze the correlation between RNFL microcirculation and clinical parameters. Discrimination capabilities of the flow metrics were assessed with the area under the receiver operating characteristic curve (AROC).

**Results:**

Twenty normal, 26 glaucoma suspect, and 42 OAG subjects were enrolled. Eyes from OAG subjects and glaucoma suspects showed significantly lower blood flux index compared with normal eyes (*P* ≤ 0.0015). Retinal nerve fiber layer blood flow metrics showed significant correlations with visual field indices and structural changes in glaucomatous eyes (*P* ≤ 0.0123). Similar discrimination capability of blood flux index compared with RNFL thickness was found in both disease groups.

**Conclusions:**

Peripapillary RNFL vascular microcirculation measured as blood flux index by OMAG showed significant differences among OAG, glaucoma suspect, and normal controls and was significantly correlated with functional and structural defects. Retinal nerve fiber layer microcirculation measurement using OMAG may help physicians monitor glaucoma.

Glaucoma is characterized by retinal ganglion cell degeneration, characteristic changes of the optic nerve head (ONH) and retinal nerve fiber layer (RNFL), and associated visual field damage.^1–4^ The evaluation of the peripapillary RNFL thickness using optical coherence tomography (OCT) plays an important role in the diagnosis and follow-up of glaucomatous eyes,^5–8^ as well as eyes suspected of having glaucoma.^9^ Vascular dysfunction in glaucomatous eyes has gained more attention in recent years as evidence has increased supporting its correlation with the development of glaucoma.^2,10,11^ However, the evaluation of microcirculation in the peripapillary RNFL is currently unavailable because (1) most of the existing imaging technology, such as fluorescein angiography (FA), generates accumulated blood flow information from funduscopically evident ocular vascular tissues, and thus is not able to identify blood flow within RNFL, and (2) even more recently developed OCT-based angiography is not able to cover a wide peripapillary RNFL area while maintaining high sampling resolution for imaging microcirculation.

We developed the optical microangiography (OMAG) imaging technique based on Fourier-domain optical coherence tomography (FD-OCT) system with active tracking capability.^12^ Optical microangiography generates three-dimensional (3D), microscopic resolution structural images as well as vascular network images in a noncontact and noninvasive manner with high repeatability and reproducibility.^12^ By detecting the differences in the scattered light from moving particles, such as red blood cells (RBCs) in the vessels, OMAG enables the visualization and quantification of microcirculation within tissue beds in the eye.^13–15^ The OMAG signal is proportional to the blood flux,^16,17^ (i.e., number of blood cells passing through vessel cross section area per unit time; please see Supplementary Material for the explanation for the concept of OMAG signal). Thus, the quantification of retinal blood flow using OMAG is possible.

The purpose of this study was to investigate the changes of microcirculation in the peripapillary RNFL in open-angle glaucoma (OAG) and glaucoma suspect eyes using OMAG, to compare the differences in RNFL blood flow between normal, glaucoma suspect, and OAG eyes, to investigate the correlations of RNFL blood flow with functional and structural changes associated with glaucoma, and to assess and compare the disease discrimination capability of the developed blood flow metrics. To the best of our knowledge, this is the first study investigating the microcirculation in peripapillary RNFL in a wide-field using OCT-based angiography technique.

## Methods

### Subjects

This study was approved by the institutional review board of the University of Washington (UW; Seattle, WA, USA) and informed consent was obtained from all subjects before imaging. This study followed the tenets of the Declaration of Helsinki and was conducted in compliance with the Health Insurance Portability and Accountability Act.

Subjects with the diagnosis of primary open-angle glaucoma (POAG), normal tension glaucoma (NTG), glaucoma suspect, or normal optic disc region were prospectively enrolled at the UW Medicine Eye Institute. Inclusion criteria were best-corrected visual acuity of 20/40 or better, refractive error between −6.0 and +3.0 diopters (D) spherical equivalent. Exclusion criteria were significant media opacity preventing high-quality imaging, any ocular disease other than glaucoma or cataract, and previous intraocular surgeries other than uncomplicated glaucoma or cataract surgery. In addition, normal subjects were also excluded for a previous diagnosis of migraine.

The diagnosis of OAG was based on characteristic optic disc findings and an abnormal RNFL thickness on FD-OCT irrespective of glaucomatous visual field (VF) loss. The glaucoma suspects participants were selected based on the presence of suspicious appearance of the optic disc (neuroretinal rim thinning or excavation), no history of IOP greater than or equal to 21 mm Hg, without evidence of repeatable glaucomatous VF damage,^18^ and a normal RNFL thickness on FD-OCT. All subjects underwent a comprehensive ophthalmologic examination at time of enrollment, and glaucoma suspects and OAG subjects received a VF exam to determine mean deviation (MD) and pattern standard deviation (PSD). All VF were performed on Humphrey Field Analyser II (Carl Zeiss Meditec, Dublin, CA, USA), and only reliable tests were included (≤33% fixation losses, false-negative results and false-positive results). One eye from each subject was included in this study. A single eye was randomly selected and imaged if both were eligible.

Blood pressure (BP) measurements were acquired at the same visit after the OMAG scan for a subgroup of subjects for each group to calculate mean ocular perfusion pressure (MOPP). MOPP was defined as 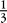 (mean arterial pressure − IOP), where mean arterial pressure = diastolic BP + 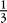 (systolic BP − diastolic BP).

### Image Acquisition and Scanning Protocol

**Figure 1:**
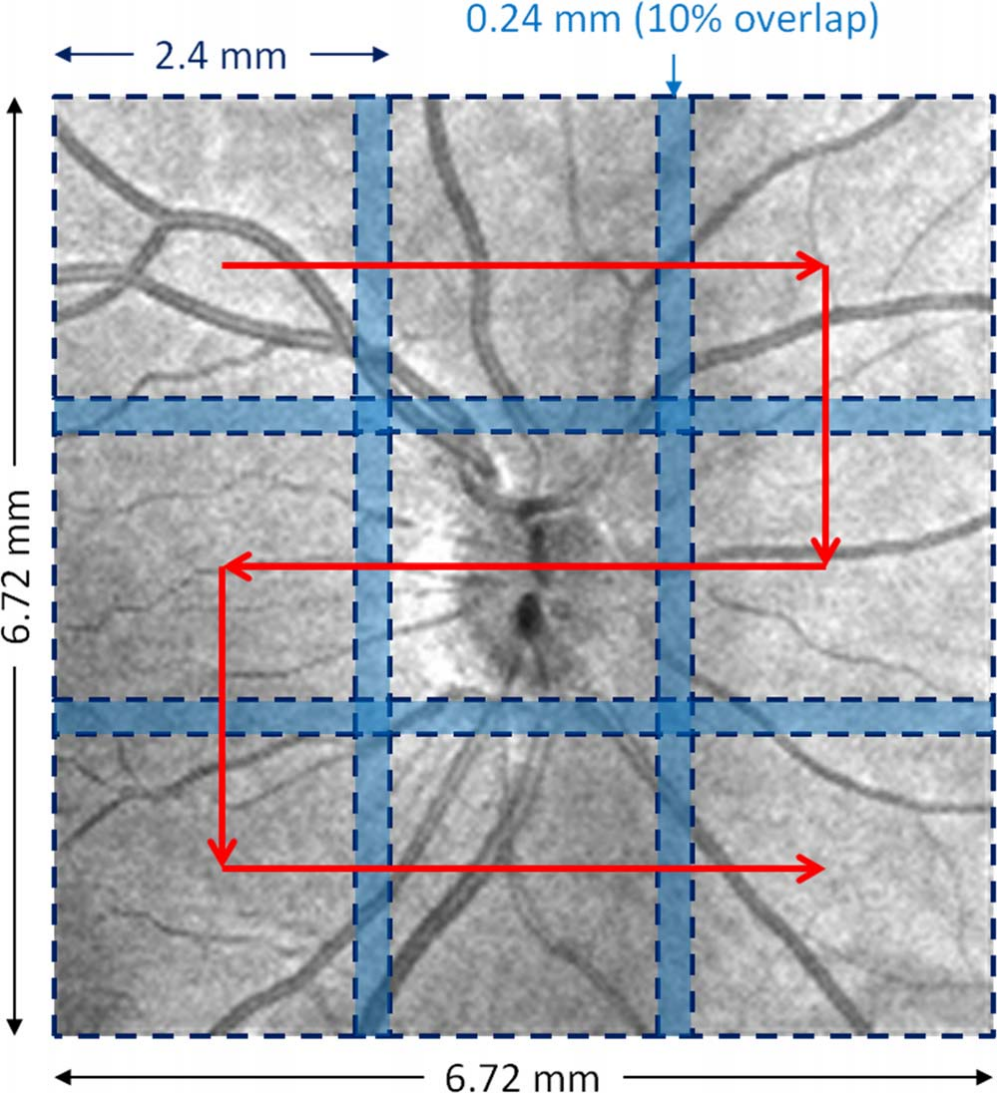
A schematic diagram indicates the location and the scanning order of the montage scanning protocol.

All the subjects were scanned with their eyes dilated and with room lighting turned off. All eyes were scanned using a 68 kHz Cirrus HD-OCT 5000–based OMAG prototype system (center wavelength at 840 nm, Carl Zeiss Meditec) with active motion-tracking capability.^12^ A 3 × 3 OMAG montage scan pattern centered at the center of the ONH was used to acquire volumetric datasets (as shown in Fig. 1). The montage scan pattern obtained nine datasets over a 6.72 × 6.72 mm^2^ area with adjacent dataset having 10% area overlap for later montaging. Each OMAG scan cube generated a volumetric dataset cover a 2.4 × 2.4 mm^2^ area. A single B-scan was composed of 245 A-scans. For each A-scan, there were 1024 sampling points collected along a 2.0-mm axial scan depth. Four consecutive B-scans were acquired at each fixed transverse location before the scanning probe proceeding to the next transverse location. A total of 245 transverse locations, located approximately 9.8-μm apart, were sampled in a 2.4-mm range. The time difference between two adjacent B-scans was approximately 3.6 ms. A proprietary motion tracking system used a line scan ophthalmoscope (LSO) to guide the OCT scans in real-time. All subsequent LSO frames were correlated with the initial LSO frame, which was set as a reference, to minimize the effect of involuntary eye movements during the image acquisition.

A montage scanning protocol was implemented under the motion tracking mode to acquire multiple cube scans. With guidance of the tracking system, the scanning probe and scanning window for multiple cube scans were automatically achieved through predefined locations (grid) on the retina (Fig. 1).

A regular OCT raster cube scan of the optic disc was also acquired using the same prototype device at the same visit to obtain a 3D structural dataset for RNFL thickness and ONH structural measurements. The scanning protocol collected 200 × 200 sampling points over a 6 × 6 mm^2^ area centered on the optic disc. Cirrus commercial software calculated the mean RNFL thickness at each point on a set-diameter (3.46 mm) circle consisting of 256 sampling points that were positioned automatically and centered at the optic disc.

The OMAG scans and the structural raster cube were considered poor quality images and omitted from further analysis if the signal strength (SS) fell below the manufacturer recommended cutoff (SS < 6) or if they showed significant eye movement. Eye movement was defined subjectively as image artifacts seen on the OCT en face images such as a horizontal frame shift larger than the average diameter of retinal vessels or a distorted oval appearance of the ONH.

### OMAG Processing

All the acquired volumetric scans were processed with a complex OCT signal-based OMAG algorithm to extract the structural and blood flow signals. The details of the OMAG algorithm were described elsewhere.^19^ In brief, the phase and intensity signals were extracted from the original complex OCT signals by fast Fourier transformation and then processed with a phase compensation method to remove the residual motion artifacts. After that, the differences between consecutive B-scan pairs at each transverse location were calculated. The average of the differences for all the repetitions at each transverse location was calculated to generate the blood flow signals, as described in Equation (1):

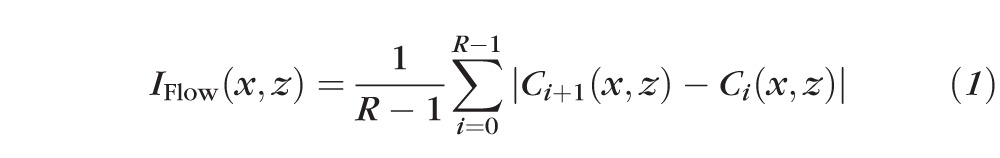
where *i* is the index of the repeated time of B-scans at each transverse location, *C*(*x*,*z*) indicates the complex OCT signal at *x*th A-scan and *z*th sampling point in the axial direction, and *R*( = 4) is the number of repeated B-scan.

All the blood flow signals were then projected onto a two-dimensional (2D) feature space based on both the structure and flow information to separate the static tissue and flow signals.^19^ By using a predetermined classification map in the feature space from the training dataset, the “false flow” signals resulted from the static background signals, such as system noise, or stability in scanning mechanism, were efficiently eliminated while the flow signals were successfully preserved, leading to enhanced contrast and signal-to-noise ratio in the final flow images.^19^

### Retinal Layer Segmentation

**Figure 2:**
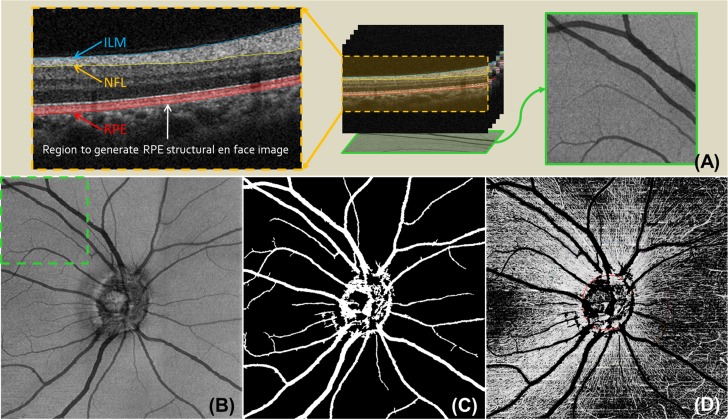
Example images for retinal layer segmentation and big retinal vessel removal. (**A**) Cross-sectional image superimposed with segmented retinal boundaries and schematic figures of how the structural en face image for RPE was created; (**B**) structural en face image for RPE, intensity averaging within 10 pixels above the detected RPE; (**C**) detected big retinal vessels; and (**D**) RNFL vascular en face image with big retinal vessels removed.

A semiautomatic retinal layer segmentation algorithm was applied to the structural OCT image to precisely separate the OCT signals into different retinal layers by detecting the gradient of OCT signals.^20^ Three boundaries were segmented: the inner limiting membrane (ILM), the posterior boundary of RNFL, and the RPE (as shown in the cross-sectional image in Fig. 2A). The ILM and posterior boundary of RNFL defined the RNFL.

The segmentation results from structural images were applied to OMAG vascular images to obtain the microvascular image within RNFL. Maximum projection analyses, which detected the signal with highest flow intensity value along each A-scan, were performed within RNFL to generate vascular en face images for RNFL. The optic disc margin was manually delineated from the structural en face image by identifying the end of Bruch's membrane and to locate the center of the ONH for further analyses.

### Big Retinal Vessels Detection

Because the RNFL is supplied by the microcirculation coming from the retinal radial peripapillary capillaries, big retinal vessels were removed when quantifying the retinal microcirculation in the peripapillary RNFL. To detect and exclude the big retinal vessels, a structural en face image for RPE, *S*_RPE_(*x*,*y*) (Figs. 2A, 2B), was generated by averaging the structural signal within 10 pixels above the detected RPE boundary for each A-scan (the red shaded area in the cross-sectional image in Fig. 2A). The high scattering and absorption properties of blood within big retinal vessels give rise to vessel shadows or dark areas in the regions below them, which could be observed from the corresponding cross-sectional images (Fig. 2A). An adaptive local thresholding based on the method proposed by Phansalkar et al.^21^ was applied to determine the threshold for each pixel on *S*_RPE_(*x*,*y*) and generated a retinal vessel map (Fig. 2C). In this study, the settings of the parameters for fine-tuning local threshold *p*, *q*, *k*, and *r* were 2.0, 12.0, 0.135, and 0.5, respectively. The size of the local neighborhood was 61 × 61 centered at each pixel.

### Retinal Blood Flux and Vessel Area Density Measurements

Because OMAG signal is proportional to the blood cells flowing through the vessels, we developed a metric to quantify the blood flow based on blood flux concept. To quantify the microcirculation in the peripapillary RNFL, we evaluated the RNFL blood flux index and vessel area density within an annulus centered at the ONH (2.5 mm as the inner diameter and 3.7 mm as the outer diameter) with the exclusion of big retinal vessels (Fig. 2D).

A multiscale Hessian filter was developed based on the method of Frangi et al.^22^ to detect blood vessels from the vascular en face images by converting the structural curvature information into probability-like estimates of vesselness.

After the vessel detection, the peripheral RNFL blood flux index and vessel area density were measured using Equation 2:

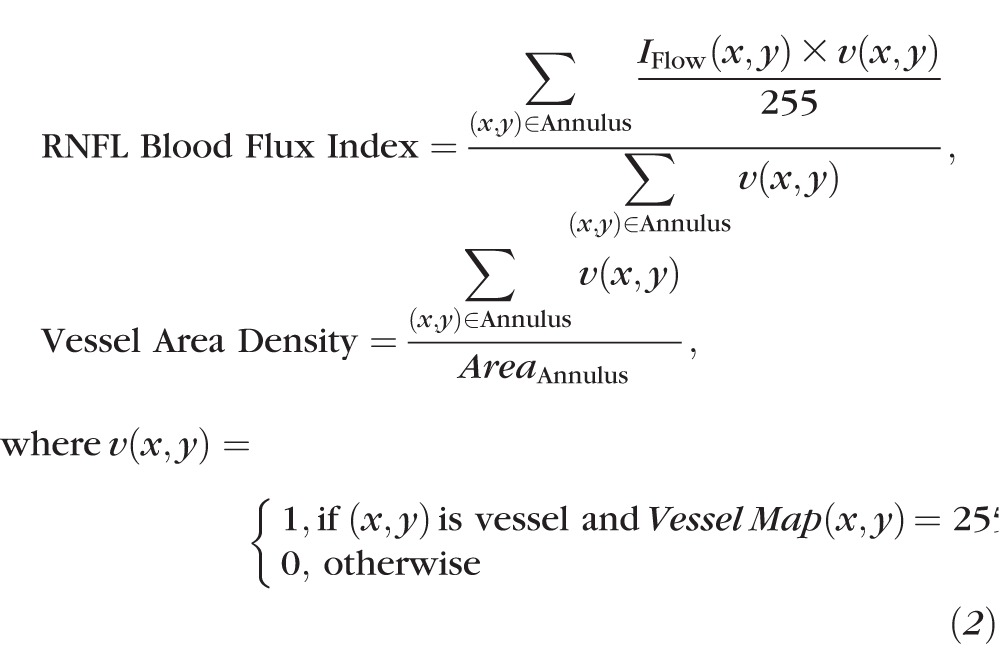



In Equation 2, for RNFL blood flux index calculation, the blood flow signal was normalized to between 0 and 1 by dividing the full dynamic range of blood flow signal intensity, and thus presented a unitless ratio. In the vessel area density calculation, the *Area*_Annulus_ indicated the number of pixel in the annulus centered at the ONH.

The peripheral RNFL blood flux index and vessel area density were measured for the entire annulus, as well as for temporal (316°–45°), superior (46°–135°), nasal (136°–225°), and inferior (226°–315°) quadrants following the way commercial system measures the RNFL thickness in four quadrants. The left eyes were mirrored to the right eye for quadrant measurements.

### Reproducibility Evaluation

An additional four normal subjects were recruited to test the reproducibility of OMAG blood flux index and vessel area density measurements with big retinal vessel removal in the peripheral RNFL. Two visits of each subject were within 6 weeks. The coefficient of variation (CV) was calculated to evaluate the reproducibility.

### Statistical Analysis

One-way ANOVAs were used to detect if there were any differences in the peripapillary RNFL blood flux index and vessel area density among normal, glaucoma suspect, and OAG eyes for the entire annulus and quadrant measurements. *P* less than 0.05 was considered statistically significant for ANOVA. Furthermore, multiple individual comparisons were conducted between each two groups via independent, 2-sample *t*-tests. For individual comparisons, Bonferroni adjustment was applied to keep the overall Type I error maintain at 5%, and therefore, for each individual comparison, *P* less than 0.0167 was considered as statistically significant. In addition to the comparisons among diagnosis groups, the differences in RNFL microcirculation between POAG and NTG groups were also investigated using independent, 2-sample *t*-tests. Linear- regression models were further used to investigate the correlation between RNFL blood flux index, vessel area density, and RNFL thickness, rim area, cup-to-disc ratio (CDR), and visual field indices. *P* less than 0.05 was considered statistically significant.

The capability of the blood flow metrics of discriminating normal from glaucomatous eyes and from glaucoma suspect eyes were assessed according to the area under the receiver operating characteristic curve (AROC), and compared with the discrimination ability of peripapillary RNFL thickness. The *P* values of multiple AROC comparisons were adjusted based on Bonferroni correction.

## Results

Twenty eyes from 20 normal subjects, 26 eyes from 26 glaucoma suspects, and 42 eyes from 42 OAG subjects (including 21 POAG and 21 NTG subjects) were enrolled. Among them, 11 normal subjects, 19 glaucoma suspects, and 14 OAG subjects had BP measurements on the same day as the OMAG scan. Table 1 summarizes the demographic information and structural clinical measurements. No significant differences were detected in age, IOP, systolic BP, diastolic BP, and MOPP among three groups (*P* ≥ 0.15, 1-way ANOVA). The average VF MD of glaucoma suspect and OAG subjects was −0.15 ± 1.26 and −5.26 ± 6.46 dB, and the average VF PSD was 1.61 ± 0.29 and 5.71 ± 4.06 dB, respectively (*P* ≤ 0.0002). For the structural measurements, significant differences were detected in all the OCT biometric parameters (mean RNFL thickness, rim area, and CDR) among normal, glaucoma suspect, and glaucoma groups (*P* < 0.0001, Table 1). Table 2 summarizes the differences in OCT biometric parameters between the groups. Both normal eyes and glaucoma suspects showed statistically significantly thicker RNFL thickness compared with glaucomatous eyes (*P* < 0.0001), but no significant difference was detected between normal and glaucoma suspects (*P* = 0.0326, *t*-test with Bonferroni adjustment).

**Table 1:**
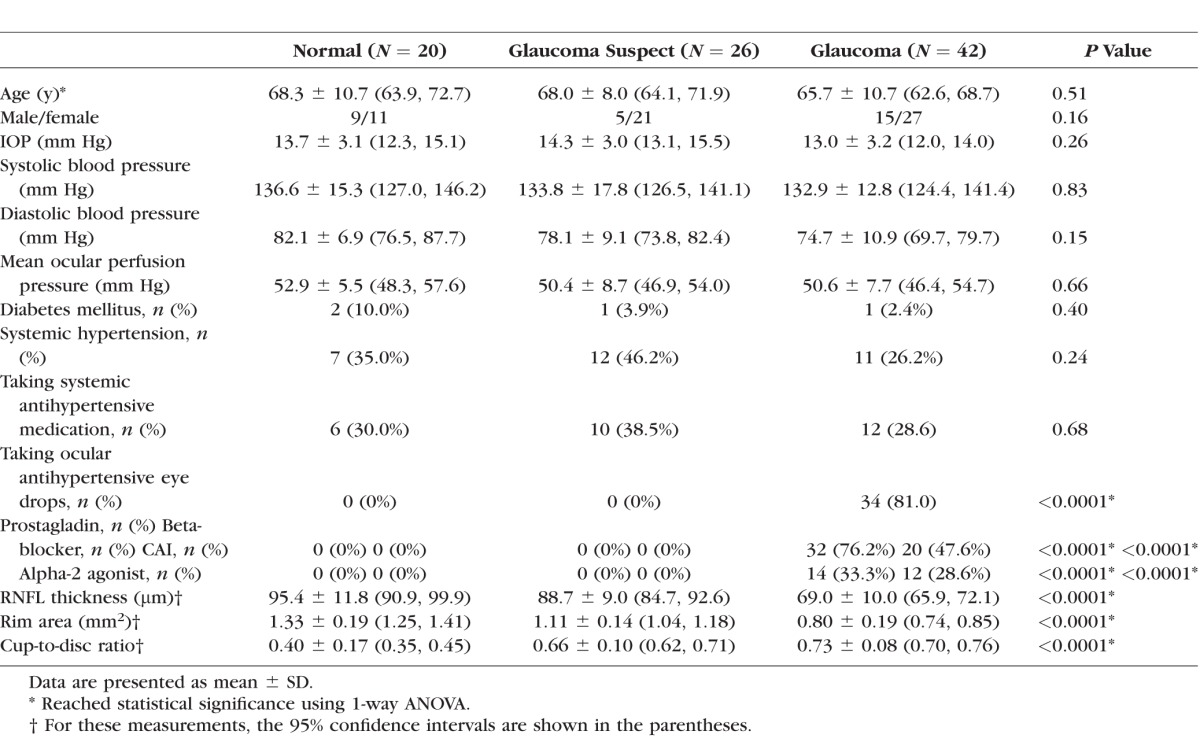
Baseline Demographic Information and OCT Biometric Parameter in RNFL and ONH Measurements

**Table 2:**
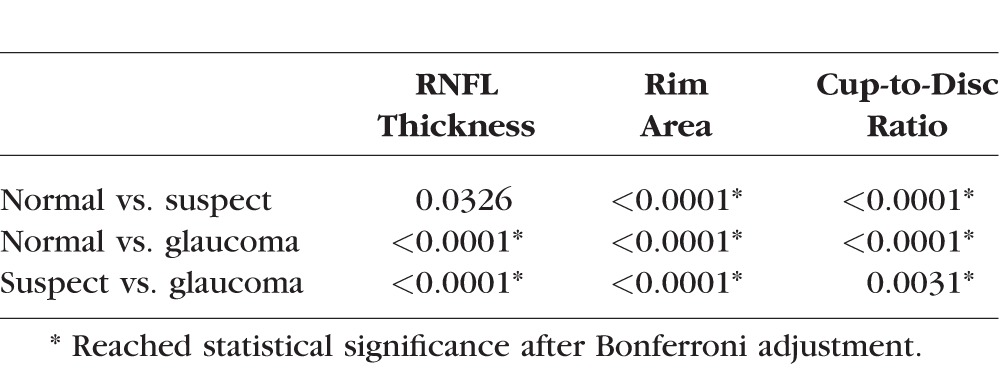
Statistical Analysis of OCT-Measured Biometrics Between Each Diagnosis Group

**Figure 3:**
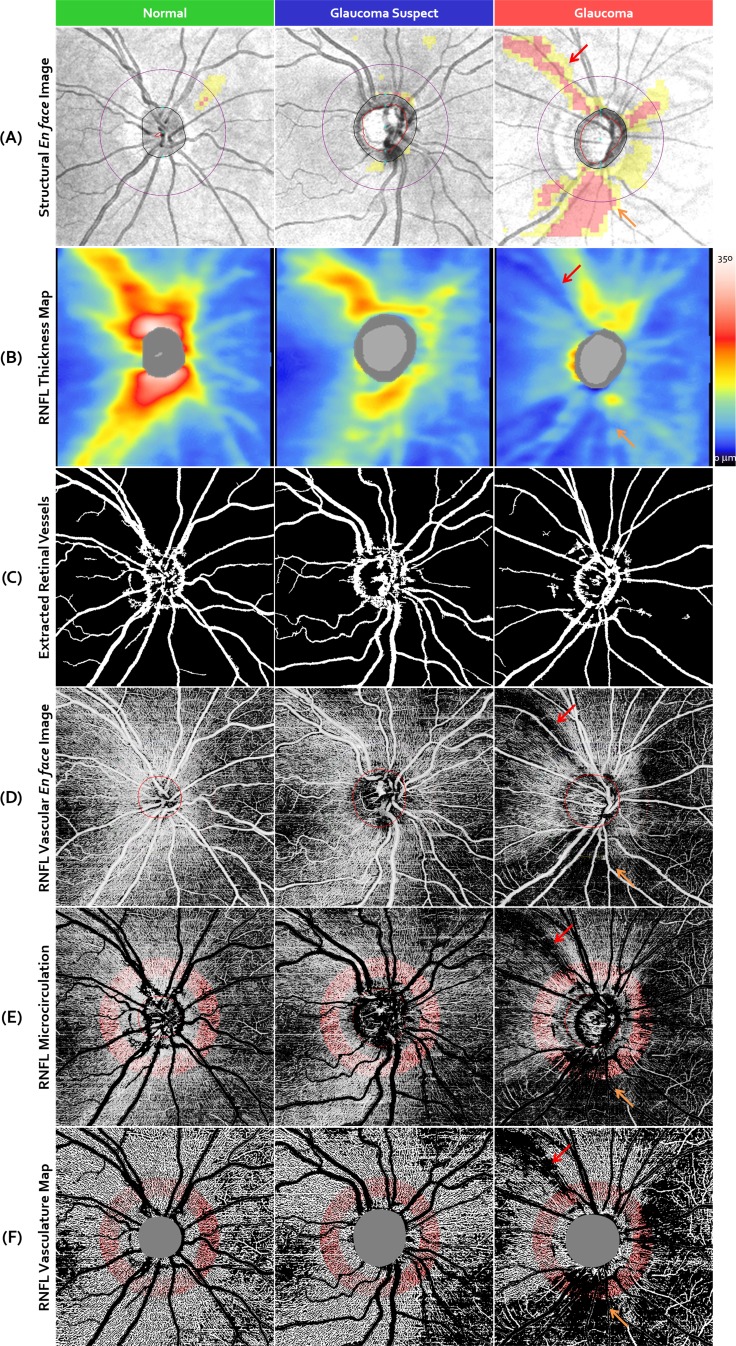
An example result of a normal, a glaucoma suspect, and a glaucomatous eye. Structural en face image with Cirrus native RNFL thickness deviation maps (**A**), RNFL thickness map (**B**), extracted retinal vessels (**C**), RNFL vascular en face image (**D**), RNFL microcirculation (**E**), and RNFL vasculature map (**F**). In the Cirrus native RNFL thickness deviation maps, areas with RNFL thickness thinner than fifth percentile of the normative database were marked in *yellow*, while RNFL thickness thinner than first percentile of the normative database were marked in *red*. The *red* and *orange arrows* in the structural en face image and RNFL thickness map indicate a nerve fiber bundle defect in superior temporal region and a defect in the inferior quadrant in the glaucomatous eye. The defects can also be observed in RNFL vascular en face image, RNFL microcirculation, and RNFL vasculature map.

Figure 3 presents exemplar results of the vascular en face images in peripapillary RNFL for a normal eye, a glaucoma suspect, and an OAG eye. The vascular en face images (row D in Fig. 3) presented a combination of major retinal vessels distribution in the RNFL as well as the retinal radial peripapillary capillaries. Comparing the structural en face image superimposed with Cirrus native RNFL thickness deviation map (row A), RNFL thickness map (row B), vascular en face image (row D), RNFL microcirculation (row E), and the vasculature map (detected vessels, row F) of the glaucomatous eye, good spatial correspondence of microcirculation reduction area and focal nerve fiber bundle defects in superior temporal region (red arrow) and a large defect in the inferior quadrant (orange arrow) were observed. Furthermore, comparing the RNFL blood flux index in the retinal capillaries among normal, glaucoma suspect, and glaucomatous eyes, a reduction in the microcirculation in glaucoma suspect and glaucomatous eyes compared with normal eyes was detected, with glaucomatous eyes showing severe reduction.

Four eyes from four normal subjects were recruited for the reproducibility test. The CV of reproducibility of blood flux was 3.6% (range, 1.0%–6.5%) for global flux index, and 2.8% (range, 1.3%–4.1%), 4.5% (range, 0.9%–7.3%), 3.0% (range, 2.1%–4.9%), and 5.5% (range, 2.0%–11.1%) for temporal, superior, nasal, and inferior quadrants. For the reproducibility of vessel area density, the CV was 2.2% (range, 0.7%–4.6%) for global vessel area density, and 1.2% (range, 0.5%–2.3%), 1.5% (range, 0.6%–3.9%), 6.8% (range, 3.1%–12.5%), and 1.1% (range, 0.1%–2.2%) for four quadrants, respectively.

The global and quadrant retinal blood flux index and vessel area density in RNFL for normal, glaucoma suspect, and OAG eyes, and comparisons between each group are summarized in Tables 3 and 4, respectively. For the global analyses, statistically significant differences were detected in blood flux index and vessel area density among three groups (*P* < 0.0001, ANOVA) and between each groups (*P* ≤ 0.0015) except for the vessel area density between normal and suspect (*P* = 0.44). For the quadrant analyses, OAG eyes showed significantly lower blood flux index and vessel area density in all four quadrants compared with normal eyes except for nasal quadrant measured as vessel area density. Glaucoma suspects showed significantly lower blood flux index in temporal quadrant compared with normal group (*P* ≤ 0.002).

**Table 3:**
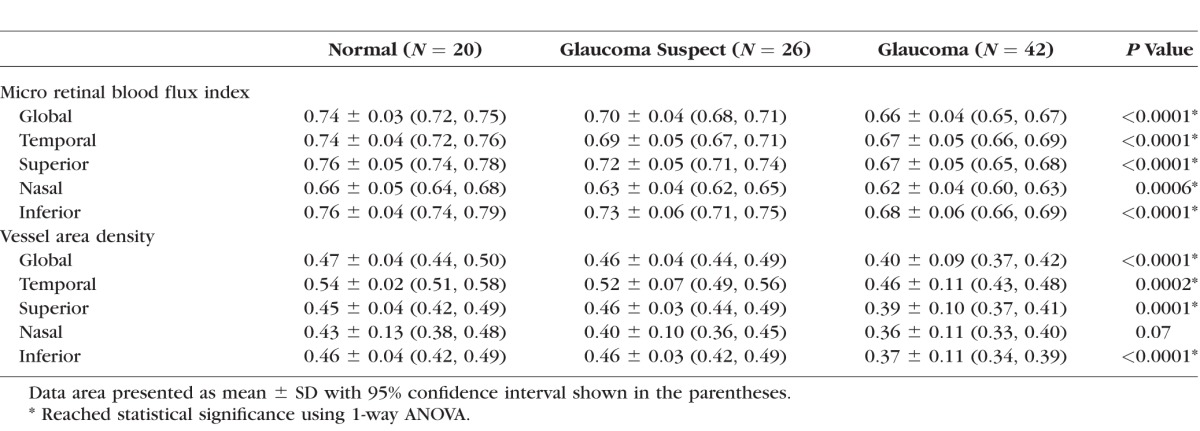
Blood Flux Index and Vessel Area Density in Peripapillary RNFL Among Normal, Glaucoma Suspect, and Glaucoma Groups

**Table 4:**
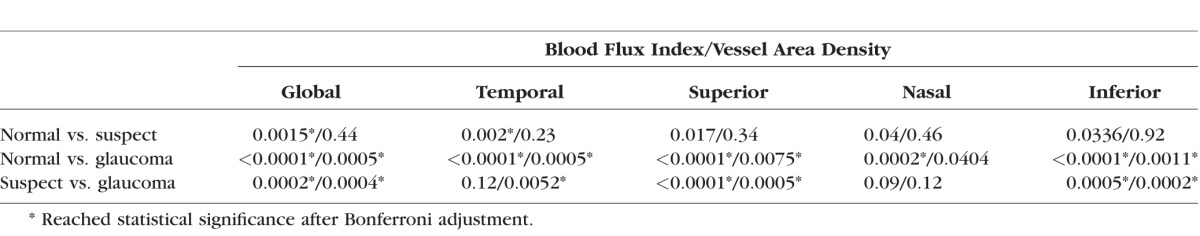
Statistical Analysis of Peripapillary RNFL Microcirculation Measurements (Blood Flux Index and Vessel Area Density) Between Each Diagnosis Group

For the blood flow metrics comparisons between POAG and NTG, no significant difference was detected in global blood flux index or in global vessel area density (data not shown; *P* > 0.39). Similar results were found in quadrant analysis: neither blood flux index nor vessel area density showed significant differences between POAG and NTG in all four quadrants (data not shown; *P* > 0.19).

Table 5 presents the findings of univariate regression analyses between blood flux index, vessel area density, and functional and structural measurements for the OAG group. Global flux index and global vessel area density were statistically significantly correlated with VF MD, VF PSD, RNFL thickness, rim area, and CDR (*P* ≤ 0.0123).

**Table 5:**
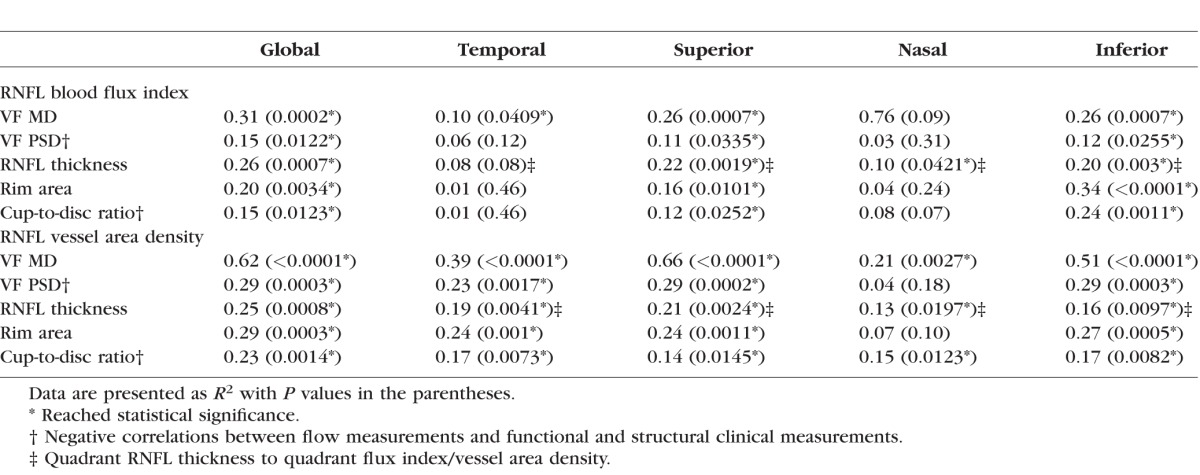
Summary of Univariate Regression Analyses Between Blood Flux Index and Vessel Area Density, and Other Functional and Structural Clinical Measurements for the Glaucoma Group

Significant correlation was detected between blood flux index and RNFL thickness in glaucoma suspect (*P* = 0.032, univariate regression analyses data not shown). No significant correlations were detected using univariate regression analyses between blood flow metrics and RNFL thickness, rim area, and CDR in the normal group (*P* ≥ 0.18; data not shown) except for between blood flux index and RNFL thickness (*P* = 0.032; data not shown).

**Figure 4:**
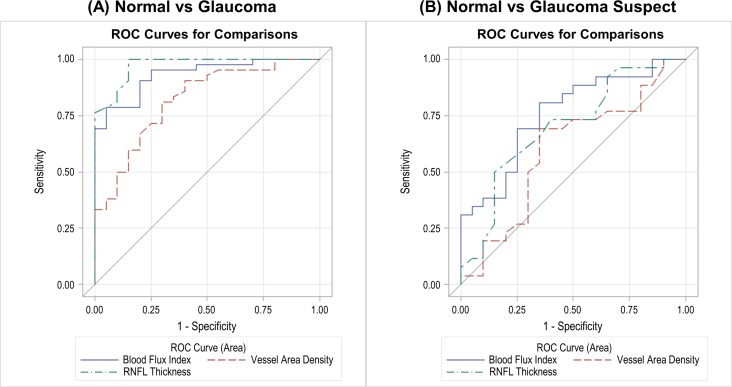
Results of AROC between (**A**) normal and glaucoma, and (**B**) normal and glaucoma suspect.

The AROCs for discriminating normal eyes from glaucomatous eyes were highest with peripapillary RNFL thickness (0.97, Table 6), followed by global blood flux index (0.93), then by vessel area density (0.82; Fig. 4A). No significant difference was detected in the discrimination ability between normal and glaucoma between RNFL thickness and blood flux index (*P* = 0.50) and between blood flux index and vessel area density (*P* = 0.06). Significant differences were found between RNFL thickness and vessel area density (*P* = 0.009). For discriminating normal eyes from glaucoma suspect, blood flux index showed the highest AROC (0.76), followed by RNFL thickness (0.70), and then by vessel area density (0.60; Fig. 4B). No significant differences were found among all three metrics (*P* > 0.10).

**Table 6:**
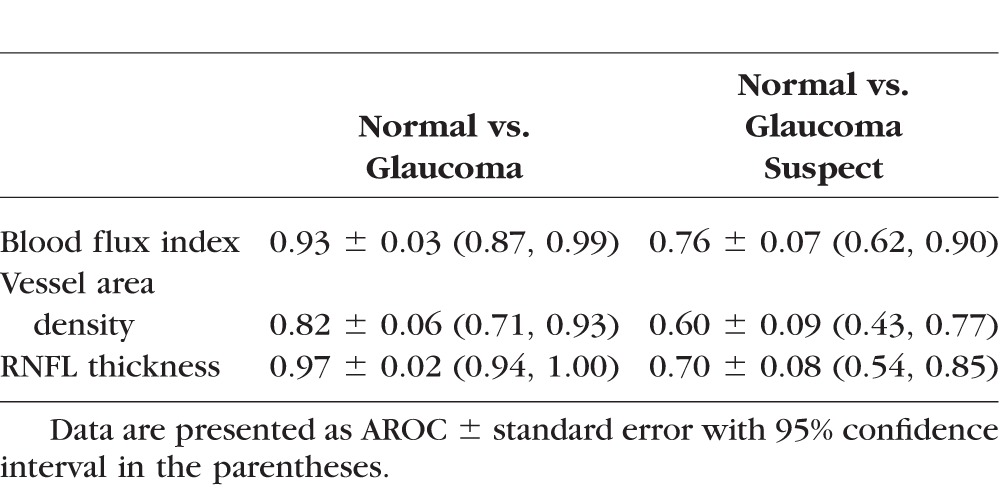
Summary of the AROC With Blood Flux Index, Vessel Area Density, and RNFL Thickness for Glaucoma and Glaucoma Suspect

## Discussion

In this study, we investigated the retinal microcirculation in the peripapillary RNFL among normal eyes, glaucoma suspects, and OAG eyes using OMAG. The active tracking system integrated in the system enabled visualization of the retinal microcirculation with a wide field of view without sacrificing imaging resolution. Significant reductions in retinal blood flow, in terms of blood flux index and vessel area density, were detected in glaucoma suspect (blood flux index only) and OAG eyes compared with age-matched normal controls. Retinal blood flux index and vessel area density showed significant correlations with visual field indices and OCT biometrics. Furthermore, blood flux index showed similar diagnostic accuracy for glaucoma and glaucoma suspect compared with the peripapillary RNFL thickness.

Our measurements of flux index and vessel area density, as with all currently published methods of OCT angiography, are unitless. This is conceptually more abstract than other attempts to measure intraocular vascular flow, like Doppler OCT (DOCT), which evaluates volumetric flow in units of microliter per minute. Although the intensity of the flow signal calculated by OMAG technique reflects and is related to the product of the number of particles (RBCs in this situation) passing through the cross-sectional area and the flow velocity, the flow signal intensity does not equal or linearly proportional to the actual blood flow flux in the retina, and there are dynamic range limitations (in terms of the detection sensitivity and saturation) of the detected flow signal. To assure the conversion equation between OMAG signal and actual blood flow flux, further investigation is warranted.

The blood flux index and vessel area density were analyzed on a 2D angiogram instead of 3D OMAG signal. Multiple advantages of a projected en face angiogram algorithm over other modalities have been described in detail by Jia et al.^23^ Additionally, we believe that our algorithms to measure blood flux index and vessel area density also have the advantages of enhanced contrast and signal-to-noise^19^; more precise segmentation and isolation of individual retinal layers, thereby providing better understanding of the regions affected by the disease. Both our unpublished data and prior studies indicate high reproducibility and reliability with OCT angiography.^23,24^ Lastly, instead of calculating the average perfusion within a fixed area across all the subjects, our flux measurements only focused on the vessel area to minimize the bias in the disc perfusion that less retinal tissue requires less microcirculation. In this way, the flux index measurements may better reflect the actual situation between healthy and diseased retinal tissues.

Focal arcuate RNFL defects showed high spatial correspondence among structural en face images superimposed with RNFL deviation maps, RNFL thickness maps, RNFL vascular en face images, RNFL microcirculation, and RNFL vasculature maps. Moreover, focal arcuate RNFL defects were easier to see in RNFL vascular en face images and vasculature maps compared with RNFL thickness maps from our subjective evaluation, which might be because of (1) OMAG's scanning pattern, with finer spatial resolution than the regular Optic Disc Cube raster scanning pattern, and (2) the ability of OMAG image processing to successfully remove the hyperreflective signals from bulk tissues, and therefore generate cleaner blood flow signals and enhance image contrasts. This indicates that OMAG may provide additional information for detecting the local RNFL defects, which otherwise would be difficult to achieve by use of other methods currently available.

Open-angle glaucoma eyes showed significantly lower peripapillary RNFL microcirculation compared with normal controls and showed significant correlations with VF indices and OCT structural measurements. Several prior studies investigated the correlations among disease severity, structural changes, and blood flow reduction in glaucomatous eyes. However, the results did not come to an agreement. Jonas et al.^25,26^ and Mitchell et al.^27^ found progressive narrowing of retinal vessels with increasing severity of glaucomatous optic neuropathy by measuring vessel diameter from fundus photos of the retina either manually or with a computer-assisted method; while Arend et al.^28^ reported no difference in retinal arterial diameter between normal control and early glaucomatous eyes using a digital scanning laser fluorescein angiography.^28^ Hwang et al.^29^ and Sehi et al.^30^ used DOCT to measure retinal blood flow and found significant differences between glaucomatous eyes and healthy controls, and significant correlation between blood flow reduction and disease severity was detected, but not between blood flow reduction and structural changes. The most relevant study evaluating peripapillary retinal perfusion (within entire retinal tissues) in glaucomatous eyes was conducted by Liu et al.^31^ using a split-spectrum amplitude-decorrelation angiography (SSADA) algorithm. The authors also detected a significant reduction in perfusion in glaucomatous eye compared with normal eyes, but found no correlation with RNFL thickness. We believe that the discrepancy is due to mismatched correlation analysis between optic disc perfusion (including surface nerve fiber layer, prelaminar tissue, and lamina cribrosa) and RNFL thickness. Optical microangiography is able to segment the retina, which enables isolation of individual layers, providing a cleaner analysis of the relationship between the microcirculation in the peripapillary RNFL and RNFL thickness, and a better understanding of the regions that are affected by glaucoma.

Even though our age-matched glaucoma suspect group had normal RNFL thickness, normal visual field, and a normal vessel area density compared with normal controls, we found a statistically significant reduction in their RNFL microcirculation compared with normal eyes. The blood flux index also showed higher AROC than the AROC with RNFL thickness when discriminating normal from glaucoma suspect, though this did not reach statistical significance. The reason for that needs further investigation, but previous studies have also detected abnormalities in blood flow in glaucoma suspects. Asejczyk-Widlicka et al.^32^ used color Doppler imaging (CDI) to show a significant decrease in end diastolic velocity in the central retina artery in a glaucoma suspect group that had normal mean RNFL thickness compared with age-matched normal controls. Piltz-Seymour et al.^33^ found significantly diminished flow in POAG suspect eyes before the development of clinically detectable visual field loss using Laser Doppler flowmetry (LDF). Similar results were found by Nicolela et al.,^34^ that impaired circulation was found by assessing the retrobulbar vessels using CDI in the less affected eyes of OAG patients who did not have any visual field loss compared with control eyes. Our results match previous findings, however, our definition of glaucoma suspect was based on the appearance of the optic disc, and morphologic changes of the optic disc could also have contributed to the size and distribution of the peripapillary vessels. With our current study, we cannot answer whether vascular dysfunction or visual field damage and/or structural tissue loss comes first when an eye is developing glaucomatous optic neuropathy. A prospective longitudinal study is needed to answer this question. We feel the OMAG technique may provide helpful and insightful information for discovering and understanding the pathophysiology of glaucoma.

Furthermore, a significant blood flux index and vessel area density reduction was detected between glaucoma suspect and OAG groups. This indicates that the blood flow metrics measured by OMAG are able to differentiate the changes in blood flow between suspect and OAG, and these may also correlate with glaucoma disease severity. All these findings may indicate the ability of microcirculation detected by OMAG to guide clinician's treatment decision in the patients with an established diagnosis of glaucoma, and also to help in the management of the glaucoma suspect patient.

We did not find significant differences in the blood flow metrics in the peripheral RNFL between POAG and NTG, neither in global measurement nor in quadrant analysis. These results are in agreement with our previous study that showed no significant difference in the ONH perfusion between POAG and NTG with similar levels of functional and structural damage using OMAG technique.^35^ Hitchings et al.^36^ compared the papillary and peripapillary circulation between POAG and NTG with similar disease severity using FA, and reported that there was no evidence of difference in circulation times between the two groups. Other studies also found similar results when comparing the ONH perfusion between POAG and NTG using various imaging techniques, such as LDF,^37^ CDI,^38,39^ and LSFG.^40^ Although vascular dysfunction has been proposed as playing a more important role for the development and progression of glaucomatous optic neuropathy in NTG than in POAG, our results may suggest vascular dysfunction is an important factor for both POAG and NTG. Further prospective study is definitely needed to reveal how vascular dysfunction affects the development and progression of POAG and/or NTG.

The AROC showed that the highest diagnostic accuracy for glaucoma was achieved with RNFL thickness, followed by blood flux index, and vessel area density; while for glaucoma suspect, the AROC was the highest with blood flux index, followed by RNFL thickness, and vessel area density. No significant differences were detected among all the comparisons except for discrimination of normal from glaucoma using RNFL thickness and vessel area density. The different behavior between glaucoma and glaucoma suspect may result from the fact that abnormal RNFL thickness was one of the inclusion criteria for our glaucoma subjects, but not for glaucoma suspects. As a consequence, RNFL thickness showed the best diagnostic accuracy when discriminating normal eyes from glaucoma eyes. Although blood flux index showed the highest AROC, to investigate whether RNFL thickness or blood flux index has the better ability to discriminate diseased and normal eyes, a prospective longitudinal study with longer observation period and larger study cohort is needed.

In the present study, we measured the microcirculation in the peripapillary RNFL within an annular area centered at the center of the optic disc in order to match the RNFL thickness measurement from the Cirrus device. As we are able to acquire montage images, it is possible to measure the peripapillary RNFL microcirculation at various locations, for example, with different diameter circle, or cover the entire 6.72 × 6.72 mm^2^ region with super-pixels to find the optimal location for glaucoma detection. Further investigation is warranted.

The unique feature of the OMAG based OCT angiography prototype system is that it is equipped with an active motion tracking system achieved by a LSO. The tracking LSO is able to guide the OCT scanning, minimize the motion artifact, and enable the montage scanning protocol for generating wide-field OMAG angiograms to visualize microcirculation in the peripapillary RNFL without laborious post processing for image stitching.^12^ All the scans are guided automatically to a predefined location, which makes post processing faster and more reliable. In addition, it allows subjects to have a break if fatigued, which dramatically reduces discomfort for a large field of view scanning.

All the enrolled subjects were scanned with their eyes dilated. However, dilation is not necessary to get good OMAG signal (unpublished data). Overall, the pupil size does not affect the signal strength of OCT signal^41,42^ as well as OMAG flow signal (unpublished data).

Our study has some limitations. First, we were not able to judge whether visual function loss leads to less demand of blood flow and thus cause the reduction in the blood flow, or whether the reduction of blood flow causes the damage of RNFL. Determining the causal relationship between blood flow and damage to the RNFL may require prospective longitudinal studies. Second, we did not obtain visual field testing in our normal subjects, though all normal subjects underwent comprehensive ocular examination and were found to have healthy optic nerves, had statistically normal peripapillary RNFL thickness, and normal optic disc measures using FD-OCT. Third, the vascular en face image was generated by maximum projection analysis, which detected the flow signal with the highest flow intensity value along the axial direction. The method was designed to be as sensitive to the slow flow signal as possible; however, it may neglect other flow signals at the same location within the same layer or pick noise signals. To reduce the noise signals, a Gaussian filter was included in the maximum projection analysis. An alternative way is to calculate the mean flow intensity along the axial direction within the RNFL, nevertheless, it may reduce the contrast and sensitivity in the resulting vascular en face image. Fourth, the medication of glaucoma subjects was not taken into account when we measured the peripapillary RNFL perfusion or performed the statistical analysis. Studies have shown that the ocular antihypertensive eye drops help decrease the IOP but may increase the blood flow in the ONH.^43,44^ Eighty percent of our glaucoma subjects were taking antihypertensive eye drops and the effects of their medications on ONH perfusion are unknown. However, there was no statistical difference in blood flow metrics between glaucoma subjects that were taking and that were not taking antihypertensive drops (*P* ≥ 0.78, *t*-tests; data not shown).

In conclusion, OMAG technique with the montage capability provides a noninvasive and noncontact method to visualize and quantify peripapillary microcirculation in specific retinal layers. Statistically significant lower RNFL blood flux index was detected in glaucoma suspect and glaucomatous eyes compared with normal controls. We found strong correlations between RNFL microcirculation and structural changes related to glaucoma damage. Retinal nerve fiber layer blood flux index measurements using OMAG may provide significant information and insight into our understanding and management of glaucoma.
